# Pastoral psychiatry – towards new understanding

**DOI:** 10.1192/j.eurpsy.2022.1404

**Published:** 2022-09-01

**Authors:** W. Kosmowski

**Affiliations:** Nicolaus Copernicus University, Department Of Psychiatry, Bydgoszcz, Poland

**Keywords:** religion, culture, pastoral psychiatry

## Abstract

**Introduction:**

Cultural psychiatry is an area of psychiatry that has been growing in importance recently. According to the new definition, mental health requires harmony with the universal values of society (Galderisi et al., 2017). Faith is considered an important factor in culture. Theology can enable a better understanding of psychiatric problems and distinction between spiritual and mental issues. “Pastoral theology aims at constructing models of redeeming activity of the Church which are current in these days, and will be current in the nearest future” (Przygoda, 2013). This discipline must recognize and evaluate the impact of contemporary sciences, including psychiatry, on theology and ecclesiastical activity.

**Objectives:**

This study aims to prepare a modern concept of pastoral psychiatry, which will be used to prepare a textbook, teaching aids and teaching plan for this discipline.

**Methods:**

Textbooks and articles in psychiatry, psychology and related disciplines, and pastoral theology monographs were analyzed. This was followed by the conceptualization of areas of interest and methodological standards.

**Results:**

Textbooks on this problem were published several decades ago (Gabriel, 1933; Bless, 1949; Polish edition issued in 1980, translated with amendments by Kaczmarek). Since then, knowledge has advanced considerably. Textbooks of psychiatry and psychology only selectively consider the Christian perspective.

**Conclusions:**

“Pastoral Psychiatry” should be helpful for priests, theologians, believers, doctors, psychologists. It requires the work of authors with theological and psychiatric competence. It will create ways of agreement, facilitate understanding of different perspectives, increase competence: theologians, priests – to better understand modern psychiatry; psychiatrists, psychologists – to better help religious patients.

**Disclosure:**

No significant relationships.

